# Implementation of a Practical Teaching Course on Protein Engineering

**DOI:** 10.3390/biology11030387

**Published:** 2022-03-01

**Authors:** Luciana C. Gomes, Carla Ferreira, Filipe J. Mergulhão

**Affiliations:** 1 LEPABE—Laboratory for Process Engineering, Environment, Biotechnology and Energy, Faculty of Engineering, University of Porto, Rua Dr. Roberto Frias, 4200-465 Porto, Portugal; luciana.gomes@fe.up.pt; 2 ALiCE—Associate Laboratory in Chemical Engineering, Faculty of Engineering, University of Porto, Rua Dr. Roberto Frias, 4200-465 Porto, Portugal; 3 Department of Chemical Engineering, Faculty of Engineering, University of Porto, Rua Dr. Roberto Frias, 4200-465 Porto, Portugal; cmferreira@fe.up.pt

**Keywords:** recombinant protein, plasmid, green fluorescent protein, protein purification

## Abstract

**Simple Summary:**

Proteins are the workhorses of the cell. With different combinations of the 20 common amino acids and some modifications of these amino acids, proteins have evolved with a staggering array of new functions and capabilities due to Protein Engineering techniques. The practical course presented was offered to undergraduate bioengineering and chemical students at the Faculty of Engineering of the University of Porto (Portugal) and consists of sequential laboratory sessions to learn the basic skills related to the expression and purification of recombinant proteins in bacterial hosts. These experiments were successfully applied by students as all working groups were able to isolate a model recombinant protein (the enhanced green fluorescent protein) from a cell lysate containing a mixture of proteins and other biomolecules produced by an *Escherichia coli* strain and evaluate the performance of the extraction and purification procedures they learned.

**Abstract:**

Protein Engineering is a highly evolved field of engineering aimed at developing proteins for specific industrial, medical, and research applications. Here, we present a practical teaching course to demonstrate fundamental techniques used to express, purify and analyze a recombinant protein produced in *Escherichia coli*—the enhanced green fluorescent protein (eGFP). The methodologies used for eGFP production were introduced sequentially over six laboratory sessions and included (i) bacterial growth, (ii) sonication (for cell lysis), (iii) affinity chromatography and dialysis (for eGFP purification), (iv) bicinchoninic acid (BCA) and fluorometry assays for total protein and eGFP quantification, respectively, and (v) sodium dodecyl sulfate-polyacrylamide gel electrophoresis (SDS-PAGE) for qualitative analysis. All groups were able to isolate the eGFP from the cell lysate with purity levels up to 72%. Additionally, a mass balance analysis performed by the students showed that eGFP yields up to 46% were achieved at the end of the purification process following the adopted procedures. A sensitivity analysis was performed to pinpoint the most critical steps of the downstream processing.

## 1. Introduction

The engineering of proteins represents a modern and powerful approach to generate novel proteins for applications in different fields as biocatalysts, therapeutic agents, and biosensors [1]. Therefore, knowledge of the basic skills of Protein Engineering is mandatory for future bioengineers and chemical engineers specialized in Biotechnology.

**Figure 1:**
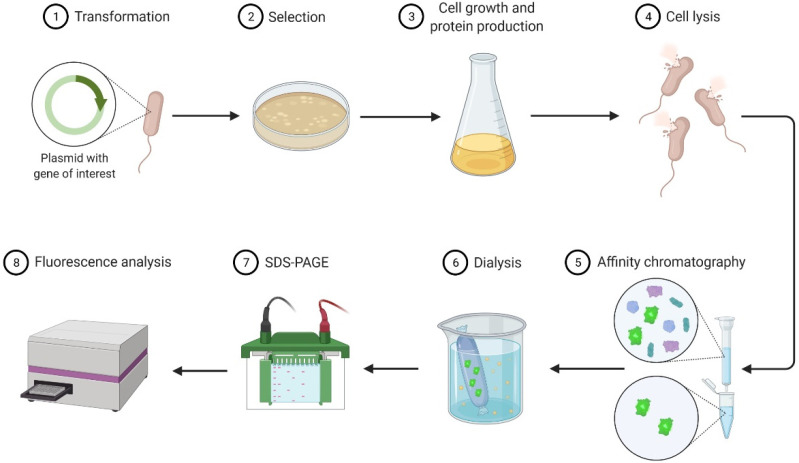
Outline of recombinant protein production and purification in *Escherichia coli*.

This work presents a description of an experimental teaching course on Protein Engineering focusing on the bacterial production, purification, and analysis of green fluorescent protein (GFP), a model recombinant protein. The process of recombinant protein production in *Escherichia coli* followed in the classes is outlined in Figure 1. The gene encoding the desired protein was first cloned into an expression vector, the plasmid vector was then transformed into an *E. coli* strain that was capable of recombinant protein production, and the transformants were grown in liquid culture [2]. Cells were recovered by centrifugation and lysed by sonication. The recombinant protein was purified by affinity chromatography and dialysed. It was then quantified and analyzed by fluorometry and sodium dodecyl sulfate-polyacrylamide gel electrophoresis (SDS-PAGE), respectively.

### 1.1. Background Theory

GFP is a small protein of about 27 kDa consisting of 238 amino acids (aa) derived from the jellyfish *Aequorea victoria* [3]. It is intrinsically fluorescent, emitting a brilliant green light when exposed to ultraviolet or blue light, due to a chromophore formed from a maturation reaction of three specific aa at the center of the protein (Ser65, Tyr66, and Gly67) [4,5]. An enhanced GFP variant (eGFP) was used in the present work, which contained substitution of Phe64 to Leu, which improves folding at 37 °C, and substitution of Ser65 to Thr that makes the protein 35 times brighter than the wild-type GFP [5,6]. The eGFP variant was obtained from the construction of a library of mutant GFP molecules using an oligo-directed, codon-based mutagenesis method [7], one of the several protein design techniques presented in the lecture course. In general, this method consists of using partially randomized synthetic oligonucleotides to generate a partially randomized gene library, expressing it in an appropriate vector to generate the protein set, and then screening the expressed proteins for improved or modified properties [8]. To obtain the eGFP, Cormack et al. [7] introduced random aa substitutions in the twenty aa flanking the chromophore Ser-Tyr-Gly sequence at aa 65–67, and then used fluorescence-activated cell sort (FACS) with a standard fluorescein isothiocyanate (FITC) filter set to screen the library for GFP mutants with increased fluorescence when excited at 488 nm. To select the fluorescence-enhanced GFP mutants, the authors started by performing a FACS scan of the total mutagenized GFP pool after isopropyl-β-D-thiogalactoside (IPTG) induction. Fluorescence emission was read with a 515/40 bandpass filter, and fluorescein and side scatter data were collected with logarithmic amplifiers. The fluorescence channel boundaries (gates) were set in the FACS scan to sort the mutant population with the highest fluorescence intensity, as described by Valdivia et al. [9]. Then, the population (events) that fell within the imposed gates was collected and amplified for a second round of FACS selection, where the gates were then defined to sort only the top 0.5% of the high fluorescent population. Finally, from this pool, 50 individual bacterial strains were compared to a control strain expressing wtGFP and it was concluded that, after induction, enhanced-GFP mutants fluoresced between 10 and 110-fold more than the control strain [7]. Many of the GFP variants were selected by visual examination using UV light, but some were selected by FACS [7,10]. A particular advantage in the use of a flow cytometer in the selection of new GFP variants is that this equipment can quantitatively detect not only the level of the GFP fluorescent signal but also spectral changes in its excitation and emission [11].

GFP-like proteins are widely used as quantitative genetically encoded markers for studying protein-protein interactions and cell tracking [12,13]. One of the most interesting aspects of GFP over other fluorescent tags is that the chromophore forms spontaneously and without accessory co-factors, substrates, or enzymes; it only requires the presence of oxygen during maturation [6], which means that the gene could be taken directly from *A. victoria* and expressed in other organisms as the Gram-negative bacterium *Escherichia coli* while still maintaining fluorescence.

**Figure 2:**
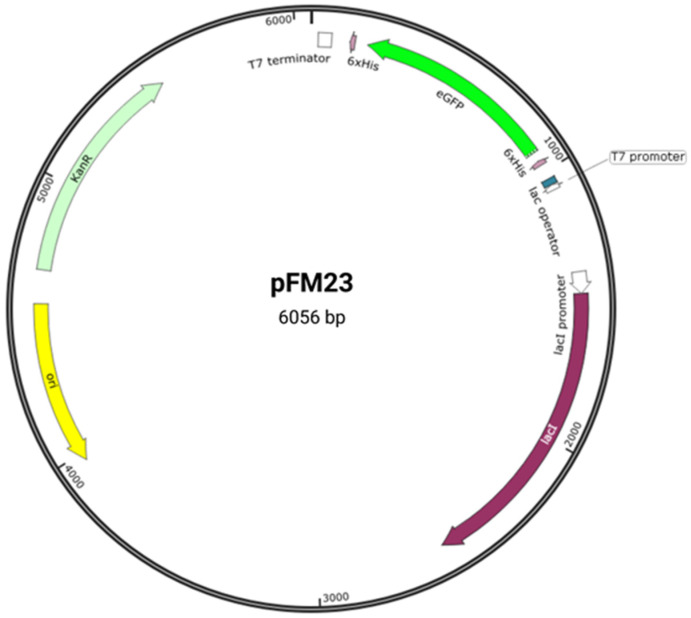
Plasmid pFM23 map. This harbours (i) a pMB1 origin of replication (ori), (ii) a repressor for the lac promoter (lacI), (iii) a transcriptional promoter from the T7 phage (T7 promoter), (iv) a lactose operator (lac operator), (v) an affinity purification tag (6 × His), (vi) a T7 transcriptional terminator (T7 terminator), (vii) a kanamycin resistance gene (KanR), and (viii) the eGFP gene (eGFP).

The heterologous expression of GFP is a particularly interesting system for didactic purposes since it can be easily observed during laboratory classes. To this end, we previously cloned the eGFP gene fused to histidine (His) tags in the pET28a vector [14], generating plasmid pFM23 (Figure 2). A linear diagram of the plasmid is presented in Supplementary Material (Figure S1), showing in detail the sequence containing the two His-tags and the eGFP gene. Plasmid pFM23 for cytoplasmic production of eGFP-His6 was constructed by digestion of plasmid pFM20 (expressing ZZ-GFP) with the NdeI/BamHI restriction enzymes and cloning of the eGFP gene into pET28A [14]. For insertion into plasmid pFM20, the eGFP coding sequence had previously been amplified from plasmid pEGFP-N1 [14]. Expression using a pET-based vector such as pET28a provides larger amounts of the target protein than other simplified systems. For this system, *E. coli* host cells engineered to carry the gene encoding T7 RNA polymerase downstream of the lac promoter are required. These cells are transformed with a plasmid that includes a copy of the T7 promoter and, adjacent to it, the gene to be expressed.

When IPTG, a lactose analog, is added to the culture medium, T7 RNA polymerase is expressed by transcription from the lac promoter [15]. The enzyme recognizes the T7 promoter on the plasmid and catalyzes the transcription of the gene of interest. T7 RNA polymerase is so selective and active that almost all of the cell resources are directed to recombinant protein expression [16]. The bacterium *E. coli* is a preferred host for the production of recombinant proteins [17,18] due to its fast growth at high cell densities, minimal nutrient requirements, well-known genetics, and availability of a large number of cloning vectors and mutant host strains [19]. This bacterium can accumulate many recombinant proteins to at least 20% of the total cell protein content [20] and translocate them from the cytoplasm to the periplasm [21]. Despite all these advantages, the expression of recombinant proteins using *E. coli* as host often results in the formation of insoluble protein aggregates called inclusion bodies [17,22]. Inclusion bodies are usually formed in the cytoplasm, and several methods have been described for the redirection of proteins from inclusion bodies into the soluble cytoplasmic fraction of cells [23]. Overall, they can be divided into procedures where protein is refolded from inclusion bodies and procedures where the expression strategy is modified to obtain soluble proteins by lowering the expression levels. For instance, this can be achieved by balancing the promoter strength and gene copy number [2,21].

After cellular disruption, several methods can be used to enrich or purify a protein of interest from other proteins and components in a crude cell lysate. One of the most powerful methods is affinity chromatography, whereby the protein of interest is purified by its specific binding properties to an immobilized ligand [24]. In this practical course, protein purification was performed by affinity chromatography of the His-tagged protein in a nickel column, followed by dialysis. His-tag expression systems are extensively used in Protein Engineering because His-tagged proteins can be easily purified by single-step affinity chromatography, namely immobilized metal affinity chromatography (IMAC), which is commercially available in different kinds of formats, the Ni-NTA matrices being the most widely used [25]. Moreover, His-tags have low molecular weight (∼2.5 kDa) and usually do not affect protein structure and function, which means that it is not necessary to separate the His-tag from the target protein [26]. Most other proteins in the lysate do not bind to the Ni-NTA resin, or bind only weakly, thus the use of His-tag and IMAC can provide relatively pure recombinant protein directly from a crude lysate.

### 1.2. Pedagogical Considerations

The practical course is offered to undergraduate bioengineering and chemical engineering students at the Faculty of Engineering of the University of Porto (Portugal). A prerequisite for attendance is basic knowledge of cellular biology, molecular biology, and microbiology. The course started in 2009 with third-year students in bioengineering and was optimized in the following years so that the procedures presented in this work were those implemented annually since 2017. This practical course is performed in six lab sessions of 3 h each where the students follow sequentially all the steps from the growth of the bacterium that expresses eGFP (Session 1) to the quantification of the purified samples (Session 6), as presented in Table 1. A number of 12–16 students organized in groups of three and four elements attended each class.

Students were familiar with the fundamentals of DNA cloning, vector design, and the pET system, since these concepts were attained in the corresponding lecture courses. For that reason, no pre-lab lecture was given, and students were expected to understand the lab work with the support of raw protocols provided by the instructors. Before starting the experimental session, a working group was selected to make a brief presentation of the theoretical concepts related to the topic of the session, as well as to present a quick protocol that was distributed to the remaining groups. Doubts were clarified and the quick protocols prepared by the remaining groups were collected. At the end of the course, the groups had access to the raw data of the other groups from the class and treated these data as a whole in the writing of their reports.

The main student learning objectives were:

- To be proficient in carrying out the following procedures: bacterial growth, cell lysis, protein purification, protein quantification, and polyacrylamide gel electrophoresis.
- To reinforce understanding of the following topics: plasmid design, recombinant protein expression, and protein purification.
- To acquire skills to operate the following equipment: UV-Vis spectrophotometer, centrifuges, sonicator, microplate reader, and electrophoresis apparatus.
- To improve the ability in critical thinking, team organization, and scientific concepts exposition and writing skills.

## 2. Materials and Methods

### 2.1. Bacterial Strain and Culture Medium

The *E. coli* strain JM109(DE3) from Promega (USA) was chosen because it is a well-characterized microorganism and is recommended for protein expression with the pET system [27]. This strain contains the expression plasmid pFM23 (Figure 2 and Figure S1 of Supplementary Material), which was previously obtained by cloning the eGFP gene into the pET28a vector [14]. The overnight cultures, as well as the growth curves (Session 1), were prepared in Luria-Bertani (LB) medium. This commercial medium is composed of 10 g/L tryptone, 5 g/L yeast extract and 10 g/L NaCl (LB-Miller, Merck KGaA, Darmstadt, Germany) and is commonly used for recombinant protein expression with the pET system [16]. For maintenance of selective pressure, the antibiotic kanamycin was added to the growth medium at a final concentration of 30 µg/mL.

### 2.2. Reagents and Equipment

Sterile or non-sterile solutions and materials were prepared by the technical assistants prior to the start of the lab sessions. The following equipment was required for the practical classes: an autoclave to sterilize the culture broth and materials, an orbital shaker set at 37 °C for bacterial culture, a UV-Vis spectrophotometer, a sterile area for microbiological manipulation, micropipettes, an ultracentrifuge, a probe sonicator, a water bath (37 and 90 °C), a shaker, a microtiter plate reader, and an electrophoresis system.

### 2.3. Session 1—Bacterial Growth Curve and Chemical Induction

#### 2.3.1. Pre-Lab Preparation

For inoculum preparation, overnight cultures of *E. coli* JM109(DE3) were grown in 100-mL shake flasks (37 °C, 120 rpm) with 25 mL of LB medium supplemented with kanamycin.

#### 2.3.2. Lab Session

On the following day, an overnight culture was attributed to each student group and its optical density (OD) was measured at 610 nm. This culture was used to inoculate 125 mL of nutrient broth with kanamycin at the starting OD_610_ value of 0.1. The OD_610_ of the culture at the zero hours was measured by removing an aliquot aseptically, and the flasks were incubated at 37 °C, 120 rpm. The previous step was repeated at 45-min intervals until the OD_610_ reached approximately 0.6–0.8 (2–3 h in LB broth at 37 °C) when each group aliquoted and frozen 1 mL of the bacterial suspension (sample A of Figure S2 of Supplementary Material). IPTG was added to the cultures at a final concentration of 0.2 mM [14].

#### 2.3.3. Post-Lab Preparation

After overnight incubation at 37 °C, 1 mL of each bacterial suspension was aliquoted (sample B of Figure S2 of Supplementary Material). The cells were harvested by centrifugation (15 min, 2744× *g*) and resuspended in 12 mL of Buffer I (50 mM Na_2_HPO_4_, 300 mM NaCl, pH 8) [14]. The resuspended cells were kept at −20 °C to be supplied to the corresponding groups in Session 2 (sample C of Figure S2 of Supplementary Material).

### 2.4. Session 2—Cell Disruption and Contact with the Chromatographic Resin

#### 2.4.1. Pre-Lab Preparation

The cells frozen in Buffer I were slowly thawed in a 37 °C water bath.

#### 2.4.2. Lab Session

A 0.5 mL aliquot of the thawed suspensions was stored (sample D of Figure S2 of Supplementary Material). The total volume of induced cells was disrupted by sonication with at least three short cycles of 15 s (Sonopuls HD 2200 probe; Bandelin Electronic, Berlin, Germany) in ice, followed by intervals of 30 s for cooling. To ensure efficient cell disruption, a cycle of freeze-thawing was then carried out, with a period of 30 min at −80 °C, followed by 30 min at 37 °C in a water bath (sample E of Figure S1 of Supplementary Material). The cell debris was removed by ultracentrifugation (15 min, 17,640× *g*, 4 °C) and the supernatant (sample G of Figure S2 of Supplementary Material) was incubated overnight with 1.5 mL of Ni-NTA resin (HisPur™ Ni-NTA Resin product no. 88221, Thermo Fisher Scientific, Carlsbad, CA, USA) at 4 °C with stirring. The pellets were resuspended in 12 mL of Buffer I and kept for later analysis (sample F of Figure S2 of Supplementary Material).

### 2.5. Session 3—Affinity Chromatography and Dialysis

The resin of each group was packed into a Fast Protein Liquid Chromatography (FPLC) column, and Buffer I was eluted (sample H of Figure S2 of Supplementary Material). The resin was washed (4-bed volumes) with Buffer I containing 20 mM imidazole. The eluent was collected for further analysis (sample I of Figure S2 of Supplementary Material). The bound eGFP was eluted in 3 mL of Buffer I containing 300 mM imidazole (sample J of Figure S2 of Supplementary Material). The resin was finally washed with 5 mL of the last buffer to ensure that all bound eGFP was collected (sample K of Figure S2 of Supplementary Material).

The eGFP eluted in the Buffer I containing 300 mM imidazole (sample J) was dialyzed overnight at 4 °C with stirring against 10 mM Na_2_HPO_4_, pH 8, and conveniently stored to be supplied to the corresponding groups in Sessions 4, 5 and 6 (sample L of Figure S2 of Supplementary Material).

### 2.6. Session 4—Total Protein Concentration

The total protein concentration of all samples collected in the previous sessions (Figure S2 of Supplementary Material) was determined by the bicinchoninic acid (BCA) assay [28]. Standard solutions of bovine serum albumin (BSA) with concentrations ranging from 0 to 500 µg/mL were first prepared from serial dilutions of a stock solution at 1000 µg/mL in Buffer I. Several dilutions of the samples were also performed (from 1:5 to 1:20) so that they could be quantified with the prepared standard curve. A stock solution of the BCA reagents (Pierce™ BCA Protein Assay Kit product no. 23225, Thermo Fisher Scientific, Carlsbad, CA, USA) was mixed as recommended by the manufacturer. Fifty µL of each standard solution or unknown concentration sample (in duplicate) were pipetted into a 96-microplate well, and 200 µL of the BCA working reagent previously prepared were added to each well. The microplate was mixed thoroughly on a plate shaker for 30 s and incubated in the dark at 30 °C for 30 min. Absorbance at 562 nm was measured using a microtiter plate reader (Synergy HT, BioTek Instruments, Inc., Winooski, VT, USA).

### 2.7. Session 5—SDS-PAGE (Sodium Dodecyl Sulfate—Polyacrylamide Gel Electrophoresis)

#### 2.7.1. Pre-Lab Preparation

An SDS-PAGE gel was prepared by the technical assistants for each group as described in detail in Table S1 of Supplementary Material. Each group knew the total protein concentration of all samples handled in Session 4 before the start of this session.

#### 2.7.2. Lab Session

About 30 µL of each sample (containing approximately 6 µg of total protein) were mixed with 10 µL of reducing solution (3 parts of Magic Mix and 1 part of β-mercaptoethanol; Magic Mix contains 10 mL of 20% SDS (sodium dodecyl sulfate), 5 mL of 1 M Tris-HCl pH 6.9, 5 mL of glycerol and 10 mg of bromophenol blue) in a 1.5 mL plastic tube. The mixtures were boiled at 90 °C for 5 min. The SDS-PAGE gel was mounted in the electrophoresis apparatus and Tris-glycine electrophoresis buffer was added to fully cover the gel. Twenty µL of each of the samples were loaded in a predetermined order into the bottom of the wells. Then, 10 μL of Precision Plus Protein Unstained Standards (ref. 161-0363, Bio-Rad, Hercules, CA, USA) were loaded in each gel run. The electrophoresis apparatus was attached to an electric power supply and a voltage of 8 V/cm was applied to the gel. After the dye front has moved into the separating gel, the voltage was increased to 15 V/cm and the gel was run until the bromophenol blue reached the bottom of the separating gel (about 3–4 h).

During the gel run time, students were asked to prepare new gels for use in other classes.

#### 2.7.3. Post-Lab Preparation

The glass plates were removed from the electrophoresis apparatus and carefully separated with a spatula in order to collect the gel. The SDS-PAGE gels were stained overnight with Coomassie Brilliant Blue (staining solution containing 0.1% Coomassie Brilliant Blue, 10% acetic acid and 40% ethanol) and destained the following day with a solution composed of 5% acetic acid and 20% ethanol. The gels were photographed.

### 2.8. Session 6: eGFP Concentration

Volumes of 100 μL purified eGFP standards with concentrations ranging from 0 to 18.3 µg/mL were prepared from a stock solution at 3.66 mg/mL in Buffer I. Regarding the samples of unknown eGFP concentration (identified in Figure S2 of Supplementary Material), 100 μL of several dilutions were prepared (from 1:4 to 1:400) to be sure that all samples could be quantified with the prepared standard curve. The samples were placed in 96-well plates and Buffer I was added to a final volume of 200 µL. Fluorescence of eGFP standards and samples was measured using a microtiter plate reader (Synergy HT, BioTek Instruments, Inc., Winooski, VT, USA) with an excitation filter of 488 nm and an emission filter of 507 nm [14].

## 3. Results and Discussion

The practical teaching course in Protein Engineering was offered to classes of 12–16 students, typically divided into working groups, each composed of three or four students. In our practical examples, we present a work schedule and results from a class of 16 students divided into four groups (G1, G2, G3 and G4).

### 3.1. Bacterial Growth

**Figure 3:**
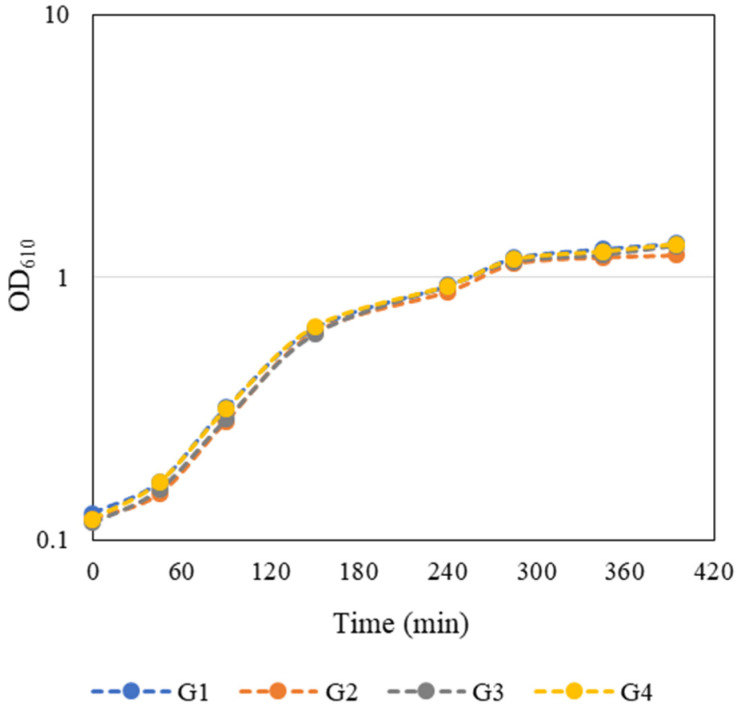
Student-generated growth curves of *E. coli* JM109(DE3) harbouring the pFM23 plasmid. After 180 min of incubation, IPTG was added to the culture medium.

An overnight culture of *E. coli* JM109(DE3) containing the pFM23 plasmid was given to each group. To start the bacterial growth curves, the OD_610_ of this stationary phase culture was determined, and a dilution factor was calculated such that by adding fresh 125 mL of LB medium the final OD_610_ would be approximately 0.1. *E. coli* growth curves presented in Figure 3 were constructed by measuring the OD_610_ every 45–60 min during class and in the first hours after class. The growth curves were very similar and the groups considered that the exponential phase of bacterial growth occurred between 90 and 285 min. Growth kinetics parameters such as maximum growth rate (*µ_max_*) and doubling time (*t_d_*) (Table 2) were then calculated separately for each individual growth curve through the logarithmic representation of the exponential part of the growth curve (Figure S3 of Supplementary Material). Regression analysis of this experimental data was performed using a Microsoft Excel spreadsheet. The slope of the line that best fits the points corresponds to *µ_max_* of each independent growth, whereas *t_d_* was estimated by Equation (1):

(1) $$t_{d}=\frac{\ln\left(2\right)}{\mu_{max}}\;.$$

An average *µ_max_* and *t_d_* of (0.00650 ± 0.00020) min^−1^ and (106.8 ± 3.3) min, respectively, were obtained. Looking at Table 2, it is possible to conclude that the values obtained from the regression analysis were similar between the working groups, with around 94% of the values fitting the linear model.

After 180 min of incubation (Figure 3), when bacterial cultures were in the exponential growth phase, IPTG was added to the culture medium. In the present work, recombinant protein expression was achieved through the transcription of the eGFP gene, which is under the control of T7 promoter in a pET-based vector (Figure 2). When bound to IPTG, the lac repressor lacI empties the lacUV5 promoter, enabling *E. coli* to transcribe the T7 gene 1, encoding the T7 RNA polymerase. The T7 RNA polymerase is then able to activate the promoter on the expression vector and transcribe the recombinant gene [15,16]. A slight reduction in cell growth of induced cultures was observed between 180 and 240 min, possibly due to the metabolic drain of biosynthetic precursors, energy, and other cellular components for plasmid replication and recombinant gene transcription. This negative effect of IPTG induction has been demonstrated for plasmid-bearing cells by several authors over the past few decades [15,29,30].

### 3.2. Protein Quantification and Analysis

After eGFP extraction and purification (Sessions 2 and 3), the total protein content in samples collected during these steps (sample G to L; Table 3 and Figure S2 of Supplementary Material) was first determined by the BCA assay. The BCA is a colorimetric method whose principle is that proteins can reduce Cu^2+^ to Cu^+^ in an alkaline solution (the biuret reaction), resulting in a purple color formation by bicinchoninic [28]. Thus, the amount of Cu^2+^ that is reduced is proportional to the amount of protein present in the solution. Bovine serum albumin (BSA) was used by the students to generate a standard curve against which unknown samples can be compared (Figure S4 of Supplementary Material). The concentration of eGFP in the same samples (G to L) was also quantified by fluorometry using a calibration curve constructed from a purified eGFP solution of known concentration (Figure S5 of Supplementary Material). Although the slope values of BCA or eGFP calibration curves were in the same order of magnitude for all groups, some variation between them was inevitably present due to pipetting errors in preparing standard solutions and loading the microplate for absorbance or fluorescence readings. However, all working groups were careful to validate their calibration curves using previously acquired knowledge of analytical chemistry [31].

The ability to predict bioprocessing performance is crucial for the production of recombinant proteins of therapeutic and prophylactic importance, especially on an industrial scale [32]. In an attempt to approach a real-world scenario of large-scale protein production, students were asked to examine the efficiency of the unit operations involved in the extraction and purification of the recombinant protein under study. For this, a full mass balance analysis was performed taking into account the concentrations of total protein and eGFP determined by the BCA and fluorometry methods, respectively, and knowing the total volume collected for each sample of the extraction and purification steps. The mass of total protein and eGFP of each sample, as well as its degree of purity (i.e., the percentage of eGFP in total protein), are summarized in Table 4 for each working group. As expected, the purity of the samples G (before the chromatography) was low compared to the other samples, varying between 14% and 24%, except for Group 4. Ideally, from the chromatography process, three samples with low protein purity should be obtained—samples H, I, and K—since they correspond to the discharges of the washing steps of the chromatographic columns. In fact, for all working groups, samples H and K had the two lowest levels of eGFP. In the case of sample I, since it corresponded to the wash fraction (unbound proteins and other compounds), it was expected that the mass of the target protein and, consequently the purity, would be residual, which was not verified. This may have resulted from an underestimation of the amount of total protein by the BCA method and/or the loss of His-tagged protein during sample loading and wash. Sample J corresponds to the eluted eGFP, thus it is expected that, like sample L collected after dialysis, it has a high degree of purity. This was verified in two of the groups (G1 and G4), with percentages of purity above 71% after chromatography. During the elution step, freedom of choice was given to the students concerning the volume in which they must collect and how they should do it (using continuous or intermittent flow with the collection of fractions at different times), always having in mind the visual aspect of the eluate and chromatographic resin. This introduces variations in the affinity chromatography protocol, which may justify the significant differences in the total and target protein content between groups. Nevertheless, the final sample of the purification process (sample L) was the one with the highest degree of purity for all working groups. Some purity values were greater than 100%, probably due to the uncertainty of the analytical methods involved in these calculations (BCA and fluorescence assays) and/or human errors (imprecision of pipetting and miscalculation, among others).

An alternative way of assessing the quality of the purification process is to determine the specific protein activity, which corresponds to the eGFP fluorescent signal per mass of total protein.

**Figure 4:**
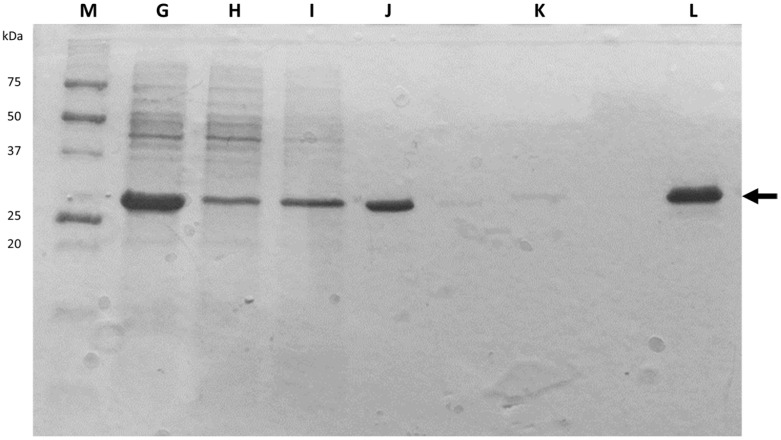
Representative SDS-PAGE gel of the samples collected between cell lysis and protein dialysis (G to L). Lane M corresponds to Precision Plus Protein unstained standards (ref. 161-0363, Bio-Rad). The arrow indicates the bands corresponding to eGFP.

To qualitatively assess the purity and relative molecular mass of proteins in the samples, polyacrylamide gel electrophoresis (SDS-PAGE) was used. This technique, associated with Coomassie blue staining, can detect bands containing as little as 100 ng of protein in a simple and relatively rapid manner (just a few hours) [33]. After reduction and denaturation by SDS, proteins migrate in the gel according to their molecular mass, allowing detection of potential contaminant and proteolysis events. Therefore, these gels provide a useful diagnostic tool for estimating the degree of purity and quality of the recombinant protein throughout the purification steps. Figure 4 shows a photograph of a representative SDS-PAGE gel where samples from G to L were loaded, as well as the molecular weight marker in the first well on the left (M). By comparing the marker bands, it is possible to determine that the stronger and better-defined bands correspond to protein(s) with molecular weights slightly higher than 25 kDa. Given that this value is very close to that found in the bibliography for eGFP (27 kDa) [3], it can be concluded that this protein has been present since the beginning of the chromatography (sample G) in relevant quantities until the post-dialysis moment, where the presence of only one band of its molecular weight revealed that it was correctly isolated from the remaining proteins (sample L). This qualitative analysis corroborated the purity results previously described and presented in Table 4.

**Figure 5:**
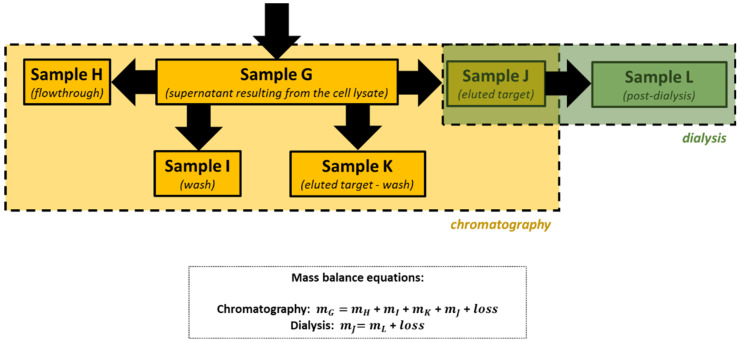
Process flow diagram and mass balance equations associated with eGFP purification.

As this is an engineering course, in addition to a full mass balance, students were also concerned with calculating the yield of each unit operation involved in the protein purification process (affinity chromatography and dialysis), as well as its overall performance (Table 5 and Figure 5). To determine the yield values presented in Table 5, each group had to consider the values of eGFP mass obtained by fluorometry (Session 6) shown in Table 4, and use Equations (2) and (3):

(2) $$\mathrm{Chromatography}\;\mathrm{yield}\;\left(\%\right)=\frac{eGFP\;mass\;in\;sample\;J\;}{eGFP\;mass\;in\;sample\;G}\times 100,$$

(3) $$\mathrm{Dialysis}\;\mathrm{yield}\;\left(\%\right)=\frac{eGFP\;mass\;in\;sample\;L\;}{eGFP\;mass\;in\;sample\;J}\times 100,$$

Concerning chromatography, yields between 34% and 72% were obtained (except for G3, whose yield was residual), which means that from the amount of eGFP mass present in sample G, it was possible to recover between 34% and 72% in sample J (eluate). The variations in results obtained between groups were most likely associated with how they decided to collect the eluate containing the protein of interest, as explained before, which can lead to higher or lower losses of eGFP. For dialysis, the yield varied between 12% and 87%. It was not expected to have high losses of eGFP in this process since it consists of a separation technique to remove small, unwanted compounds (such as imidazole and salts) from proteins in solution by selective and passive diffusion through a semi-permeable membrane. Different events may have led to the low yield determined by the students: human error (inaccuracy of pipetting or miscalculation), or technical problems associated with non-specific binding of the target protein to the dialysis membrane or protein loss due to wrong membrane pore size or lose closure of the dialysis tube. The low ionic strength of the dialysis buffer may also have caused protein precipitation. Although the dialysis membrane used in this work was made of cellulose acetate and this material is less susceptible to non-specific protein adsorption, some eGFP sticking may have occurred. One way to avoid this is to add a low concentration of a nonionic detergent such as Triton X-100 or Tween-20 to the sample and dialysis buffer in order to coat the plastic surface and any exposed hydrophobic patches of the protein. The issue of protein precipitation during dialysis can be circumvented by increasing the ionic strength of the buffer resulting in salting-in (increased protein solubility). Despite the low dialysis yield, it was possible to obtain total protein recoveries up to 46%.

These sequential laboratory experiments were successfully applied by students as they were able to extract, purify, and quantify the protein of interest (eGFP) from an *E. coli* culture containing the expression plasmid (pFM23), and finally discuss the performance of the extraction and purification procedures they learned. Moreover, students were able to assess some of the benefits of Protein Engineering techniques such as mutagenesis (yielding more active proteins) and fusion protein tagging (which enabled high-level purification in a single-chromatographic step). This engineering course gives students the opportunity to experience different techniques commonly used in the pharmaceutical industry and academia to produce recombinant proteins.

## 4. Conclusions

The course is intended to introduce bioengineering and chemical engineering students to widely used techniques in molecular biology and protein biochemistry laboratories, covering all the steps that are essential to produce recombinant proteins in *E. coli*. The pFM23 system proved to be a useful, didactic tool for demonstrating protein expression and purification. The natural fluorescence of GFP makes its visual detection possible during expression in bacteria and purification by affinity chromatography, in parallel with accurate techniques to detect it with fluorometry and electrophoresis. We believe the proposed scheme may serve as a benchmark for expressing and purifying other fluorescent proteins in Protein Engineering courses.

## Figures and Tables

**Table 1:** Planning of laboratory sessions.

Session Number	General Topic	Tasks
1	Protein expression	Bacterial growth curve and chemical induction
2	Protein extraction	Cell disruption and contact with the chromatographic resin
3	Protein purification	Affinity chromatography and dialysis
4	Protein quantification	Total protein concentration
5	Protein analysis	SDS-PAGE
6	Protein quantification	eGFP concentration

**Table 2:** Parameters obtained by regression analysis for each group.

Group	*R*^2^ *	*p*-Value **	*µ_max_* (min^−1^)	*t_d_* (min)
G1	0.9430	0.0289	0.00634	109.4
G2	0.9270	0.0372	0.00660	105.0
G3	0.9401	0.0304	0.00673	103.0
G4	0.9349	0.0331	0.00631	109.8

* *R*^2^ is the coefficient of determination and measures of how well the regression predictions approximate the real data points. ** Since the *p*-values are much lower than the significance level (0.05), we rejected the null hypothesis that the coefficient is zero.

**Table 3:** List of samples to be analyzed in Session 4, 5 and 6.

Sample Identification	Session	Content
A	1	Cell culture in the exponential phase
B	1	Grown cell culture
C	1	Supernatant resulting from the centrifugation of the cell culture
D	1	Cell pellet resuspended in Buffer I
E	2	Cell lysate
F	2	Cell debris resulting from the centrifugation of the cell lysate
G	2	Supernatant resulting from the centrifugation of the cell lysate
H	3	Flowthrough (unbound material)
I	3	Wash
J	3	Eluted target
K	3	Eluted target (wash)
L	3	Post-dialysis

**Table 4:** Mass balance and eGFP purity for each working group.

Group	Sample	Total Protein Mass (mg)	eGFP Mass (mg)	Purity (%)
G1	G	39.475	8.056	20.4
	H	29.709	1.176	4.0
	I	3.759	1.207	32.1
	J	5.146	5.325	103.5
	K	0.248	0.051	20.6
	L	2.686	3.737	139.1
G2	G	68.138	9.323	13.7
	H	54.898	1.282	2.3
	I	5.746	4.867	84.71
	J	6.868	3.169	46.14
	K	0.625	0.005	0.73
	L	2.672	2.745	102.73
G3	G	55.630	13.361	24.0
	H	51.235	1.358	2.7
	I	1.993	1.067	53.5
	J	0.580	0.227	39.1
	K	0.191	0.007	3.7
	L	1.621	2.743	169.2
G4	G	23.586	13.530	57.36
	H	7.107	2.664	37.48
	I	2.404	1.676	69.70
	J	13.856	9.791	70.66
	K	0.218	0.013	6.06
	L	1.088	1.207	110.9

**Table 5:** eGFP yield for chromatography and dialysis by each working group.

Group	Chromatography Yield (%)	Dialysis Yield (%)
G1	66.1	70.2
G2	34.0	86.6
G3	1.7	1208.4 *
G4	72.4	12.3

* This value is not physically possible since there was no production or addition of recombinant protein during the dialysis stage.

## Data Availability

The data presented in this study are available on request from the corresponding author. The data are not publicly available yet as some data sets are being used for additional publications.

## Supplementary Materials

The following supporting information can be downloaded at: https://www.mdpi.com/article/10.3390/biology11030387/s1. Figure S1: (A) Linear map of plasmid pFM23 showing in detail the (B) His-tag location and protein domain organization. Figure S2: Summary scheme of tasks to be performed in each lab section. Special emphasis was placed on the samples to be kept, properly identified with a letter from A to L, for further analysis in Sessions 4, 5 and 6. Table S1: List of reagents used for preparing an SDS-PAGE gel with a concentration of 15% in acrylamide. Figure S3: Logarithmic representation of the growth curves shown in Figure 3 to estimate the growth kinetics parameters (*µ**_max_* and *t_d_*). Figure S4: Comparison of the calibration curves obtained by the working groups (G1, G2, G3 and G4) for the bicinchoninic acid (BCA) assay. Figure S5: Comparison of the calibration curves obtained by the working groups (G1, G2, G3 and G4) for eGFP quantification.

## References

[1] Poluri K.M., Gulati K. (2017). Biotechnological and Biomedical Applications of Protein Engineering Methods. Protein Engineering Techniques: Gateways to Synthetic Protein Universe.

[2] Gomes L.C., Mergulhão F.J., Berhardt L.V. (2019). Production of Recombinant Proteins in *Escherichia coli* Biofilms: Challenges and Opportunities. Advances in Medicine and Biology.

[3] Chalfie M., Tu Y., Euskirchen G., Ward W.W., Prasher D.C. (1994). Green fluorescent protein as a marker for gene expression. Science.

[4] Zacharias D.A., Tsien R.Y. (2006). Molecular biology and mutation of green fluorescent protein. Methods Biochem. Anal..

[5] Stepanenko O.V., Stepanenko O.V., Kuznetsova I.M., Verkhusha V.V., Turoverov K.K. (2013). Beta-barrel scaffold of fluorescent proteins: Folding, stability and role in chromophore formation. Int. Rev. Cell. Mol. Biol..

[6] Stepanenko O.V., Verkhusha V.V., Kuznetsova I.M., Uversky V.N., Turoverov K.K. (2008). Fluorescent proteins as biomarkers and biosensors: Throwing color lights on molecular and cellular processes. Curr. Protein Pept. Sci..

[7] Cormack B.P., Valdivia R.H., Falkow S. (1996). FACS-optimized mutants of the green fluorescent protein (GFP). Gene.

[8] Neylon C. (2004). Chemical and biochemical strategies for the randomization of protein encoding DNA sequences: Library construction methods for directed evolution. Nucleic Acids Res..

[9] Valdivia R.H., Hromockyj A.E., Monack D., Ramakrishnan L., Falkow S. (1996). Applications for green fluorescent protein (GFP) in the study of hostpathogen interactions. Gene.

[10] Crameri A., Whitehorn E.A., Tate E., Stemmer W.P.C. (1996). Improved Green Fluorescent Protein by Molecular Evolution Using DNA Shuffling. Nat. Biotechnol..

[11] Galbraith D.W., Anderson M.T., Herzenberg L.A., Sullivan K.F., Kay S.A. (1998). Flow Cytometric Analysis and FACS Sorting of Cells Based on GFP Accumulation. Methods in Cell Biology.

[12] Zacharias D.A., Baird G.S., Tsien R.Y. (2000). Recent advances in technology for measuring and manipulating cell signals. Curr. Opin. Neurobiol..

[13] Gomes L.C., Mergulhão F.J., Berhardt L.V. (2018). Applications of Green Fluorescent Protein in Biofilm Studies. Advances in Medicine and Biology.

[14] Mergulhão F.J., Monteiro G.A. (2007). Analysis of factors affecting the periplasmic production of recombinant proteins in *Escherichia coli*. J. Microbiol. Biotechnol..

[15] Gomes L.C., Monteiro G.A., Mergulhão F.J. (2020). The Impact of IPTG Induction on Plasmid Stability and Heterologous Protein Expression by *Escherichia coli* Biofilms. Int. J. Mol. Sci..

[16] Novagen (2005). pET System Manual.

[17] Mergulhão F.J.M., Monteiro G.A., Cabral J.M.S., Taipa M.A. (2004). Design of bacterial vector systems for the production of recombinant proteins in *Escherichia coli*. J. Microbiol. Biotechnol..

[18] Sanchez-Garcia L., Martín L., Mangues R., Ferrer-Miralles N., Vázquez E., Villaverde A. (2016). Recombinant pharmaceuticals from microbial cells: A 2015 update. Microb. Cell Factories.

[19] Baneyx F. (1999). Recombinant protein expression in *Escherichia coli*. Curr. Opin. Biotechnol..

[20] Pines O., Inouye M. (1999). Expression and secretion of proteins in *E. coli*. Mol. Biotechnol..

[21] Mergulhão F.J.M., Summers D.K., Monteiro G.A. (2005). Recombinant protein secretion in *Escherichia coli*. Biotechnol. Adv..

[22] Mergulhão F.J., Taipa M.A., Cabral J.M., Monteiro G.A. (2004). Evaluation of bottlenecks in proinsulin secretion by *Escherichia coli*. J. Biotechnol..

[23] Sørensen H.P., Mortensen K.K. (2005). Soluble expression of recombinant proteins in the cytoplasm of *Escherichia coli*. Microb. Cell Fact..

[24] Urh M., Simpson D., Zhao K., Burgess R.R., Deutscher M.P. (2009). Affinity Chromatography: General Methods. Methods in Enzymology.

[25] Spriestersbach A., Kubicek J., Schäfer F., Block H., Maertens B., Lorsch J.R. (2015). Purification of His-Tagged Proteins. Methods in Enzymology.

[26] Booth W.T., Schlachter C.R., Pote S., Ussin N., Mank N.J., Klapper V., Offermann L.R., Tang C., Hurlburt B.K., Chruszcz M. (2018). Impact of an N-terminal Polyhistidine Tag on Protein Thermal Stability. ACS Omega.

[27] Yanisch-Perron C., Vieira J., Messing J. (1985). Improved M13 phage cloning vectors and host strains: Nucleotide sequences of the M13mp18 and pUC19 vectors. Gene.

[28] Smith P.K., Krohn R.I., Hermanson G.T., Mallia A.K., Gartner F.H., Provenzano M.D., Fujimoto E.K., Goeke N.M., Olson B.J., Klenk D.C. (1985). Measurement of protein using bicinchoninic acid. Anal. Biochem..

[29] Bentley W.E., Davis R.H., Kompala D.S. (1991). Dynamics of induced CAT expression in *E. coli*. Biotechnol. Bioeng..

[30] Einsfeldt K., Severo Júnior J.B., Corrêa Argondizzo A.P., Medeiros M.A., Alves T.L., Almeida R.V., Larentis A.L. (2011). Cloning and expression of protease ClpP from *Streptococcus pneumoniae* in *Escherichia coli*: Study of the influence of kanamycin and IPTG concentration on cell growth, recombinant protein production and plasmid stability. Vaccine.

[31] Homem V., Alves A., Santos L. (2014). Development and Validation of a Fast Procedure To Analyze Amoxicillin in River Waters by Direct-Injection LC–MS/MS. J. Chem. Educ..

[32] Tripathi N.K., Shrivastava A. (2019). Recent Developments in Bioprocessing of Recombinant Proteins: Expression Hosts and Process Development. Front. Bioeng. Biotechnol..

[33] Walker J.M., Walker J.M. (2009). SDS Polyacrylamide Gel Electrophoresis of Proteins. The Protein Protocols Handbook.

