# Supplementary material for: Implementation of a Practical Teaching Course on Protein Engineering
Source: Biology (Basel). 2022 Mar 1;11(3):387. doi: 10.3390/biology11030387 (PMC8944992; doi:10.3390/biology11030387)
Supplement: Supplementary file 1 [file biology-11-00387-s001.zip › biology-1583227-supplementary.pdf]

Supplementary Material

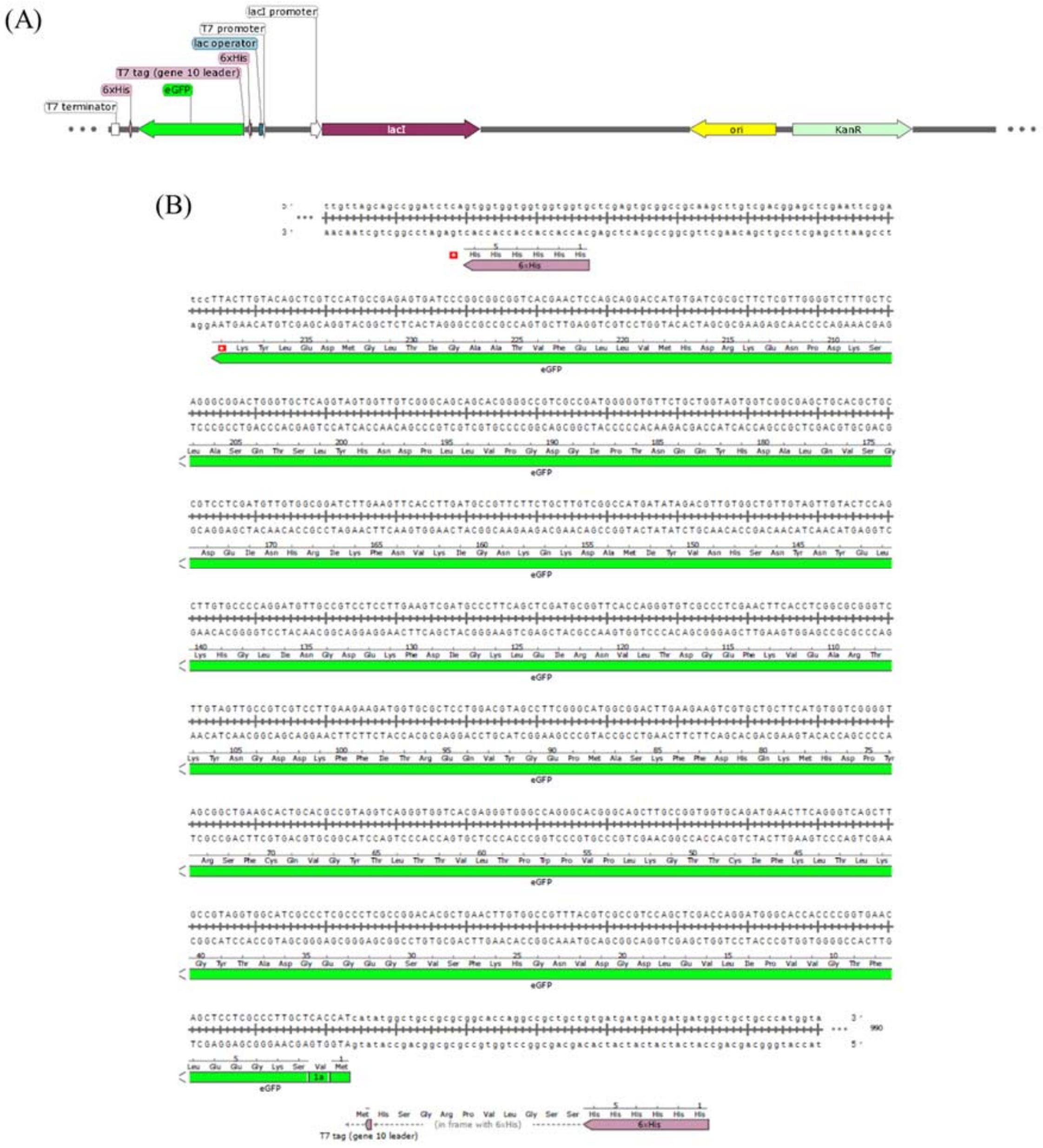

Figure S1. (A) Linear map of plasmid pFM23 showing in detail the (B) His-tag location and protein domain organization.

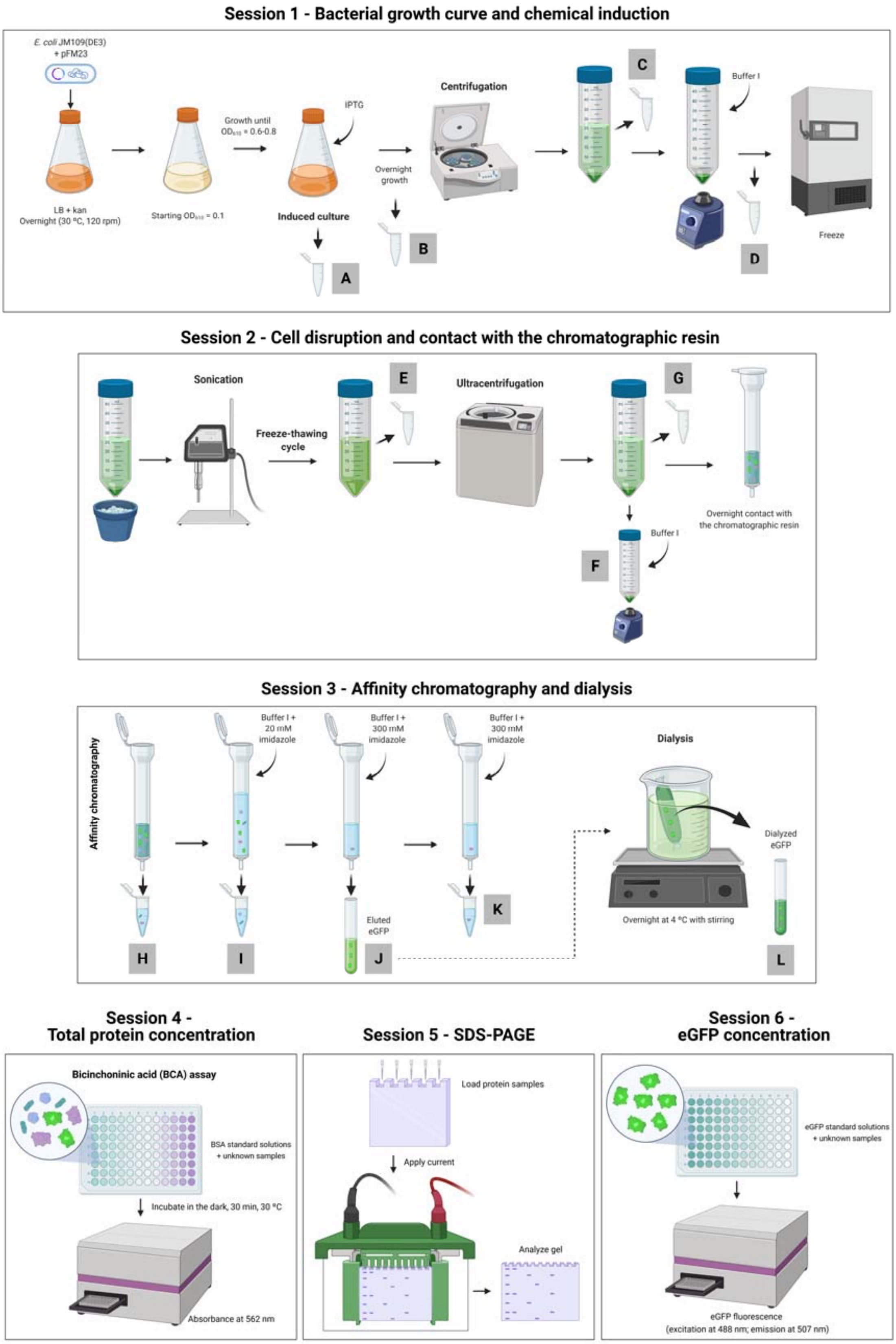

Figure S2. Summary scheme of tasks to be performed in each lab section. Special emphasis was placed on the samples to be kept, properly identified with a letter from A to L, for further analysis in Sessions 4, 5 and 6.

Table S1. List of reagents used for preparing an SDS-PAGE gel with a concentration of 15% in acrylamide

**Reagents***	**Separating gel**	**Stacking gel**
40% acrylamide	5.63 mL	1.28 mL
2% bisacrylamide	0.65 mL	0.7 mL
1 M Tris-HCl pH 8.7	5.6 mL	-
1 M Tris-HCl pH 6.9	-	1.25 mL
10% SDS	150 µL	100 µL
H_2O	1.1 mL	6.77 mL
TEMED	10 µL	25 µL
10% Ammonium persulphate	50 µL	50 µL

* All the reagents were added except TEMED and ammonium persulphate that were only mixed when all the gel apparatus was ready to cast the gel.

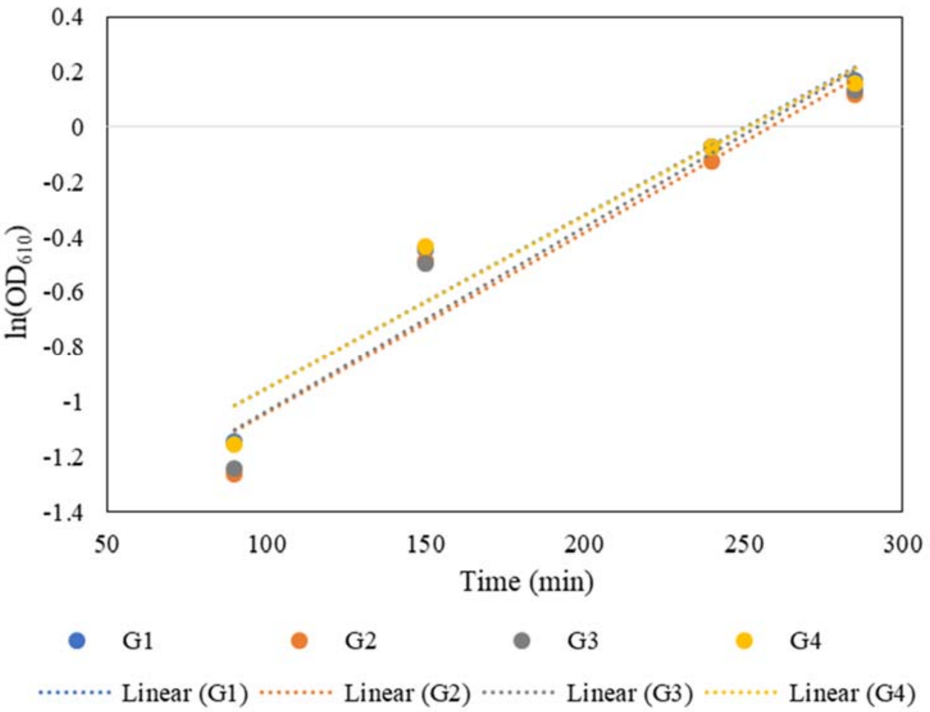

Figure S3. Logarithmic representation of the growth curves shown in Figure 3 to estimate the growth kinetics parameters (μ_{max} and t_d).

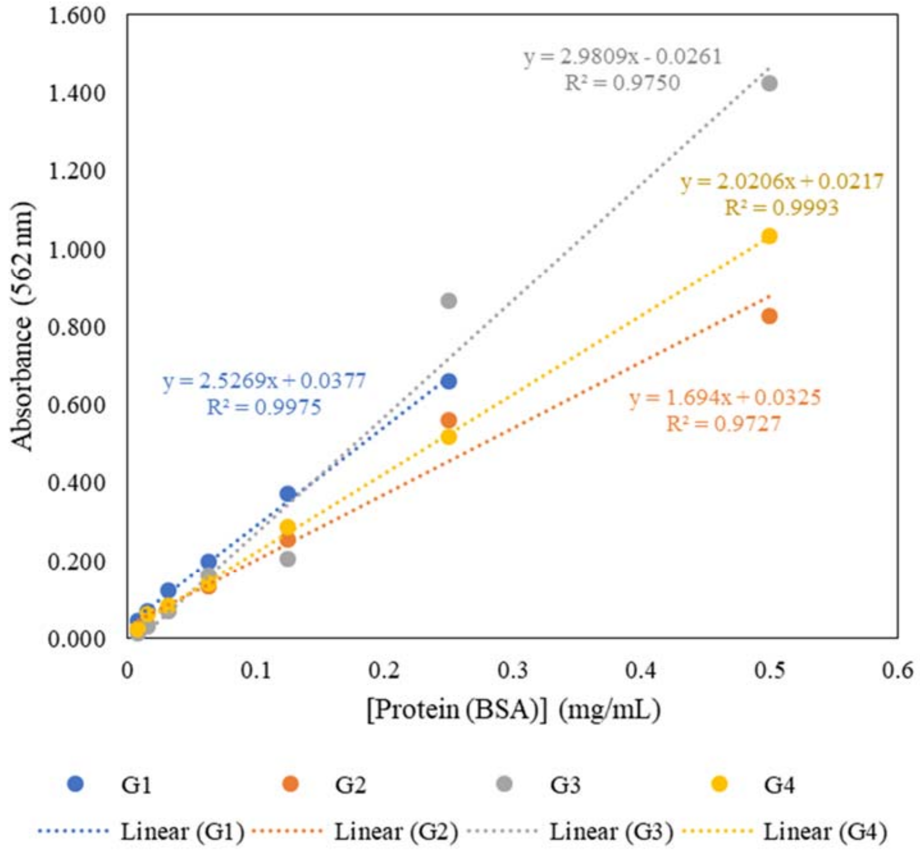

Figure S4. Comparison of the calibration curves obtained by the working groups (G1, G2, G3 and G4) for the bicinchoninic acid (BCA) assay.

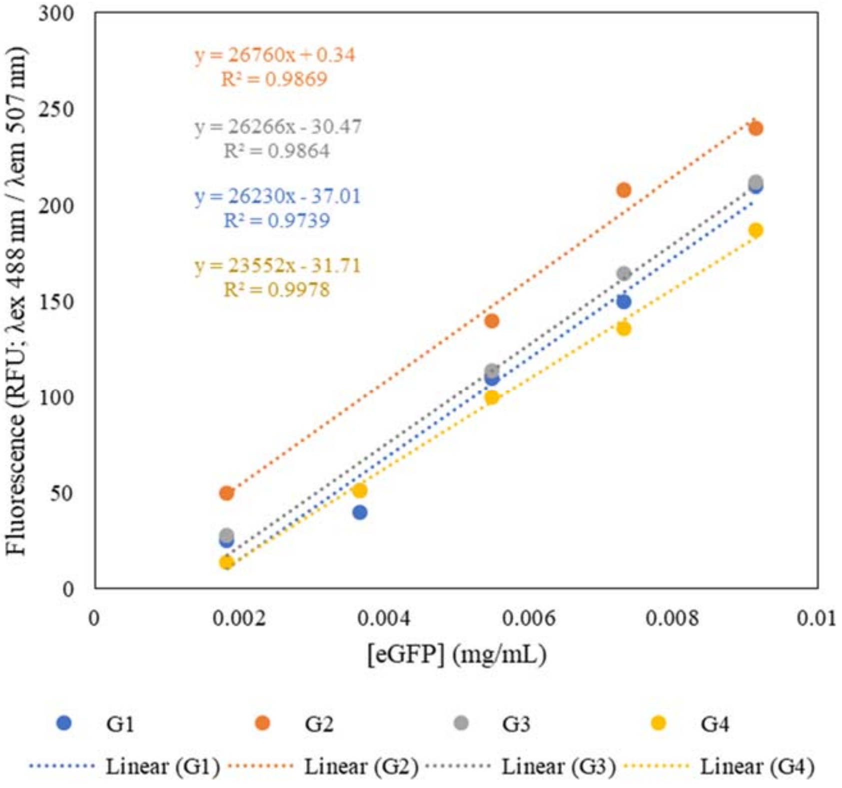

Figure S5. Comparison of the calibration curves obtained by the working groups (G1, G2, G3 and G4) for eGFP quantification.
